# Supplementary material for: Membrane Adaptations and Cellular Responses of Sulfolobus acidocaldarius to the Allylamine Terbinafine
Source: Int J Mol Sci. 2023 Apr 15;24(8):7328. doi: 10.3390/ijms24087328 (PMC10138448; doi:10.3390/ijms24087328)
Supplement: Supplementary file 1 [file ijms-24-07328-s001.zip › Supplementary Materials-Figures.pdf]

Supplementary Materials

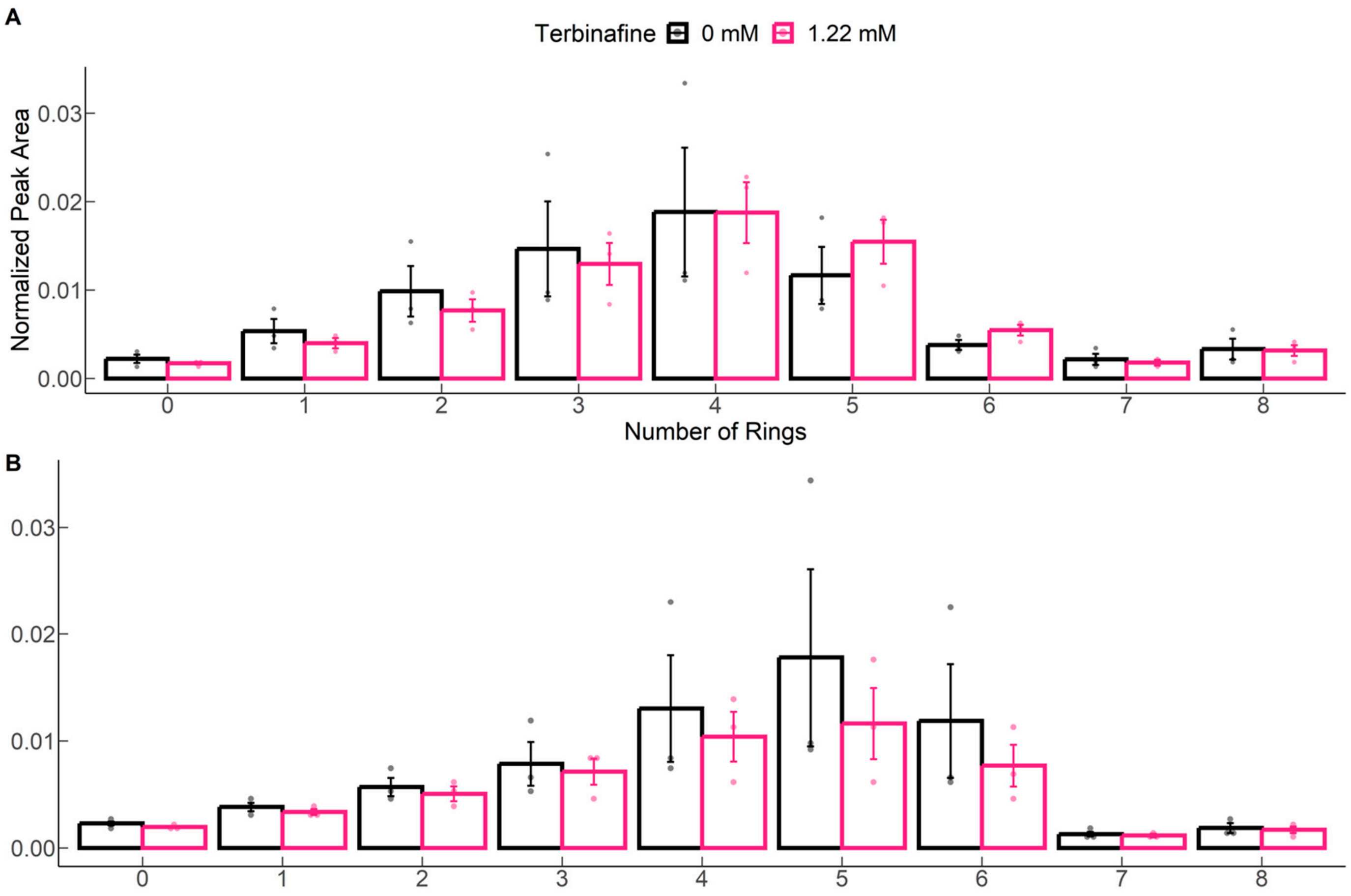

Figure S1. **Effect of terbinafine on GDGT cyclization in *S. acidocaldarius* :** Normalized peak areas for GDGT 0-8 rings in (A) Exponential phase and (B) stationary phases of growth. Error bars represent standard error of the mean. Dots represent biological replicates.

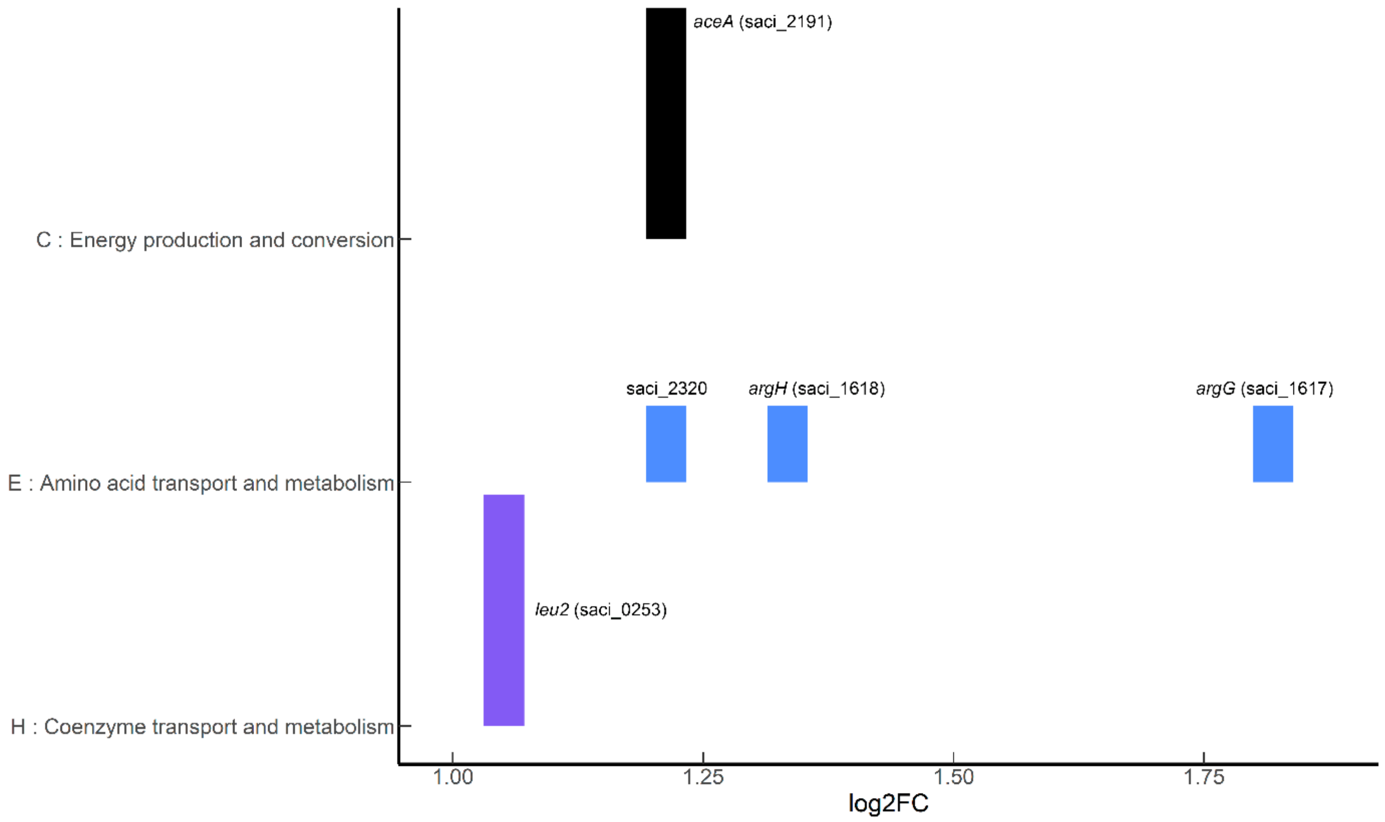

Figure S2. **arCOG mapping of significantly affected genes after growth with 0.31 mM terbinafine (p-adjusted < 0.05):** Density plot illustrates the distribution of log2 fold changes for the five genes affected by terbinafine after three hours of growth

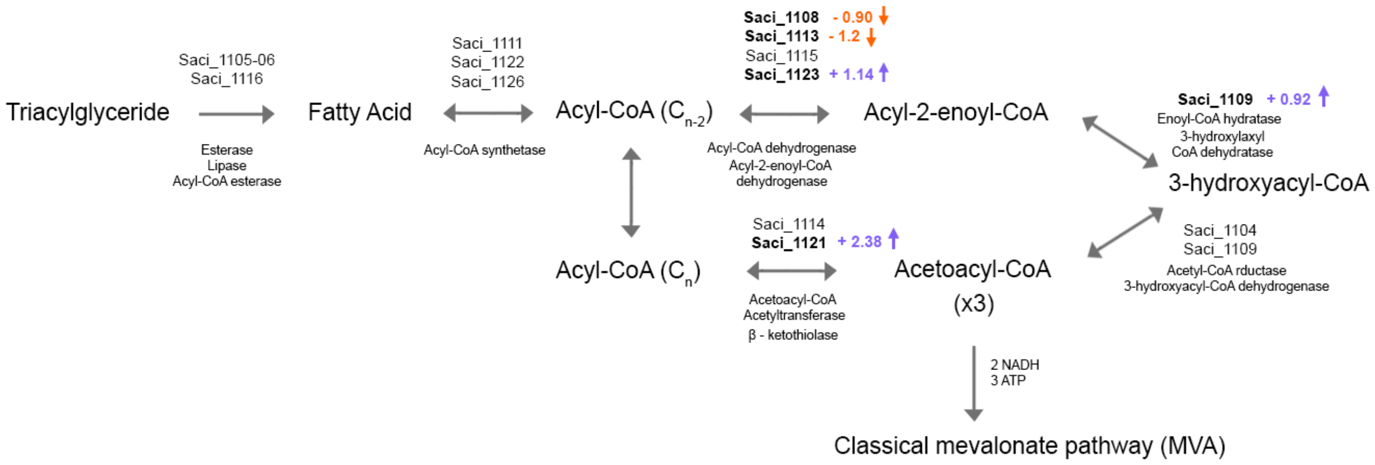

Figure S3. **Effect of terbinafine on the proposed fatty acid metabolism cluster in *S .acidocaldarius* (p <0.05):** The proposed catabolic fatty acid metabolism/lipase gene cluster in *S. acidocaldarius* is based on genomic annotations and predictions. Violet/orange arrows indicate upregulated and

downregulated genes respectively, numbers correspond to log2 fold change values. Unaffected genes are colored grey. This scheme has been adapted from (Wang et al., 2019).

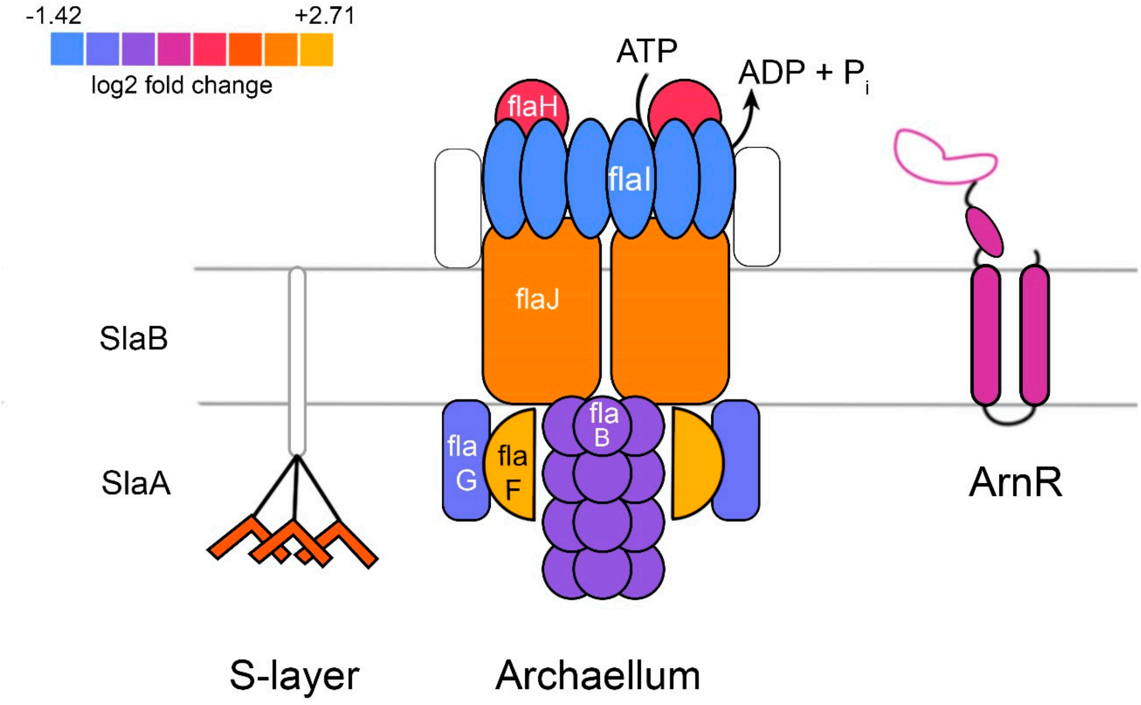

Figure S4 .Effect of terbinafine on the cell envelope of *S. acidocaldarius* (p-adjusted < 0.05): Colors represent log2fold change values in the diagram. No color signifies unaltered transcript levels.
